# Do Antibiotics Potentiate Proteases in Hemotoxic Snake Venoms?

**DOI:** 10.3390/toxins12040240

**Published:** 2020-04-09

**Authors:** Christoffer V. Sørensen, Cecilie Knudsen, Ulrich auf dem Keller, Konstantinos Kalogeropoulos, Cristina Gutiérrez-Jiménez, Manuela B. Pucca, Eliane C. Arantes, Karla C. F. Bordon, Andreas H. Laustsen

**Affiliations:** 1 Department of Biotechnology and Biomedicine, Technical University of Denmark, DK-2800 Kongens Lyngby, Denmark; chvis@dtu.dk (C.V.S.); cecknu@dtu.dk (C.K.); uadk@dtu.dk (U.a.d.K.); konka@dtu.dk (K.K.); christiarte@gmail.com (C.G.-J.); mpucca@dtu.dk (M.B.P.); 2 BioPorto Diagnostics A/S, DK-2900 Hellerup, Denmark; 3 Medical School, Federal University of Roraima, Boa Vista BR-69310-000, Brazil; 4 Department of BioMolecular Sciences, School of Pharmaceutical Sciences of Ribeirão Preto, University of São Paulo, Ribeirão Preto BR-14040-903, Brazil; ecabraga@fcfrp.usp.br (E.C.A.); karlabordon@yahoo.com.br (K.C.F.B.)

**Keywords:** snakebite envenoming, venom, antibiotics, snake venom serine proteases, snake venom metalloproteases, infection, drug-toxin interactions

## Abstract

Antibiotics are often administered with antivenom following snakebite envenomings in order to avoid secondary bacterial infections. However, to this date, no studies have evaluated whether antibiotics may have undesirable potentiating effects on snake venom. Herein, we demonstrate that four commonly used antibiotics affect the enzymatic activities of proteolytic snake venom toxins in two different in vitro assays. Similar findings in vivo could have clinical implications for snakebite management and require further examination.

## 1. Introduction

Snakebite envenoming is a serious medical emergency, which can cause a number of life-threatening toxic effects, but which can also lead to secondary infections due the introduction of pathogenic bacteria at the bite site. While the toxic effects are often treated with antivenom, the secondary infections are typically treated with antibiotics. The administration of antibiotics is recommended by the World Health Organization (WHO) for snakebite victims, with the purpose of preventing sepsis or necrosis, or avoiding secondary infection when the bite wound has obviously been tampered with [1]. Antibiotics are also sometimes administered prophylactically to snakebite victims, even under conditions different from those recommended by the WHO [2,3,4]. This study aims at investigating, for the first time, the effect of antibiotics on snake venom toxins. Specifically, it was investigated whether antibiotics may potentiate snake venom serine protease activity, as unpublished serendipitous results from our group suggest that this might be the case.

## 2. Results and Discussion

**Figure 1:**
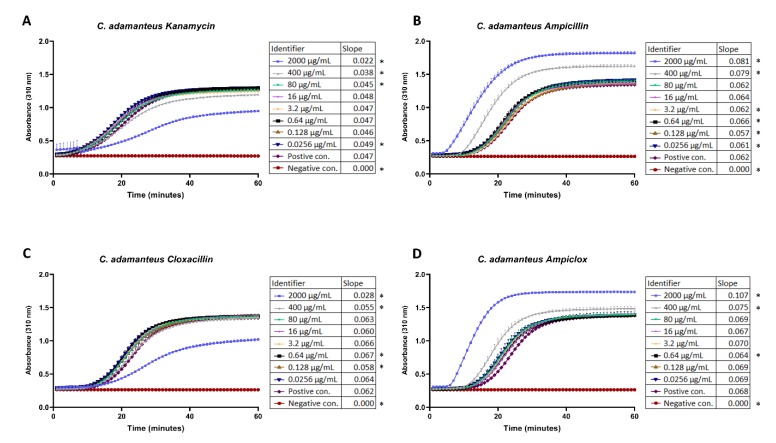
Cleavage of fibrinogen to fibrin by 8 µg/mL *C. adamanteus* whole venom in the presence of varying concentrations of kanamycin (**A**), ampicillin (**B**), cloxacillin (**C**), and ampiclox (**D**). 8 µg/mL *C. adamanteus* whole venom was used as positive control, and PBS was used as negative control. No antibiotics were added in the controls. Measurements were done in triplicate. Each point is the average of triplicates with error bars showing the standard deviation. The slopes in the legends table were calculated using linear regression in the interval 30% to 70% of the maximum activity of each slope. Asterisk (*) notes statistical difference (*p* < 0.05) compared to the positive control. Statistics were carried out by comparing each concentration to the positive control using an ANOVA, followed by Dunnett’s test.

The results from fibrinogen cleavage assays indicate that antibiotics can affect fibrinolytic activity in complex ways. At a concentration of 2000 µg/mL, kanamycin and cloxacillin inhibited cleavage (Figure 1A,C), while at the same concentration (and also at a concentration of 400 µg/mL in the case of ampicillin), ampicillin and ampiclox (prepared as a 1:1 mixture of ampicillin and cloxacillin) potentiated cleavage (Figure 1B,D). 

**Figure 2:**
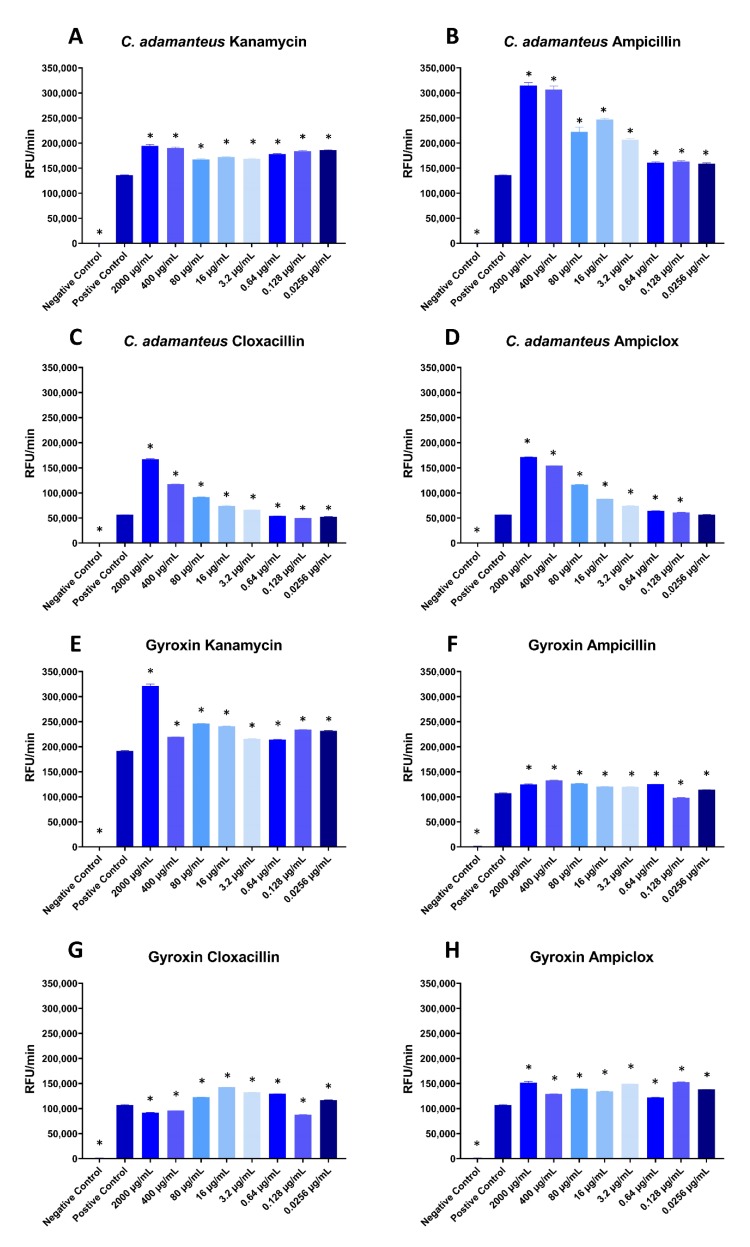
Quenched fluorescent peptide-based substrate assay monitoring cleavage of ES011 by 8 µg/mL *C. adamanteus* whole venom (**A–D**) or 6 µg/mL gyroxin (**E–H**) in the presence of varying concentrations of kanamycin (**A,E**), ampicillin (**B,F**), cloxacillin (**C,G**), and ampiclox (**D,H**). 8 µg/mL *C. adamanteus* whole venom was used as positive control and PBS was used as negative control. No antibiotics were added to the controls. RFU: Relative fluorescence units. Measurements were done in triplicate except for *C. adamanteus* with cloxacillin and ampiclox, which were carried out in duplicate. Each column represents the RFU/min calculated using linear regression with error bars showing the standard deviation. Asterisk (*) notes statistical difference (*p* < 0.05) compared to the positive control. Statistics were carried out by comparing each concentration to the positive control using an ANOVA, followed by Dunnett’s test.

Interestingly, in the fluorescence assay with *Crotalus adamanteus* (eastern diamondback rattlesnake) venom, all four antibiotics appear to potentiate cleavage at most concentrations (Figure 2A–D), with the exception of two or three of the lowest concentrations of cloxacillin and ampiclox, where the level of relative fluorescence units per minute (RFU/min) was similar to the positive control (Figure 2C,D). All tested concentrations of kanamycin and ampiclox significantly potentiated the purified snake venom serine protease gyroxin [5] (Figure 2E,H), and so did all concentrations of ampicillin, except the second lowest concentration (0.128 µg/mL), which instead showed slightly inhibiting effects (Figure 2F). The last antibiotic, cloxacillin, showed very interesting results on gyroxin (Figure 2G). Here, the two highest concentrations, together with the second lowest (2000 µg/mL, 400 µg/mL, and 0.128 µg/mL) showed inhibiting effects. The rest of the concentrations showed potentiating effects, with the largest potentiating effect being at 16 µg/mL cloxacillin.

The relationships between antibiotic concentrations and potentiation observed in Figure 2 are complex. For ampicillin, cloxacillin, and ampiclox, there is a positive correlation between antibiotic concentration and potentiation of *C. adamanteus* proteases, except for the three lowest concentrations, which have similar potentiating effects regardless of concentration. However, for kanamycin, the degree of potentiation of *C. adamanteus* proteases appears to be fairly constant, independent of the concentration of kanamycin (Figure 2A). In one case, an intermediate concentration of antibiotics (cloxacillin) had the greatest effect on proteolytic activity of gyroxin (Figure 2G).

It was hypothesized that the observed effects could be misleading due to either a pH change from the addition of antibiotics, which could have altered the catalytic activity of the enzymatic toxins, or the antibiotics changing the measurements or the background. This was tested in different control assays (Figures S1–S4). The addition of antibiotics did indeed change the pH of the sample (between 0.36 and 0.44 pH units); however, after normalizing the pH and comparing fluorescence assays with and without normalized pH, it was found that the pH change had no significant effect on the observed results (Figure S1A,B). By measuring the negative controls for three different concentrations of the antibiotics in the fluorescence assay, it was observed that for the highest and second highest tested concentrations of ampicillin, ampicillin did have a minor effect on the RFU/min (Figure S2B). However, this minor effect was disregarded as the main reason for the larger (dose-dependent) change in RFU/min observed in Figure 2B,D, as the minor increase in RFU/min was not significantly above the background signal. The negative control tests for the fibrinogen cleavage assay showed no increase in absorbance in the assay (Figure S3). Lastly, to assess whether the observed effects were genus-specific, it was also tested if the antibiotics could potentiate the activity of proteases from the venom of *Bothrops asper* (fer-de-lance) in the fluorescence assay. The results indicate that this is indeed possible, suggesting that the potentiation is not genus-specific (Figure S4).

From this study, we conclude that the tested antibiotics do have potentiating effects on the cleavage of the fluorogenic peptide substrate ES011 by *C. adamanteus* whole venom and gyroxin. However, we cannot conclude if the antibiotics selectively affect serine proteases or if multiple toxin families are affected. Other toxins, such as snake venom metalloproteases, have also been known to cleave fibrinogen and may also cleave other substrates known to be cleaved by serine proteases, such as the substrate used in the fluorescence assay. By consulting the MEROPS database [6], it was found that these types of metalloproteases exist in the *Crotalus* genus, exemplified by atroxase and ruberlysin [7,8]. Furthermore, the large variation of effects that the antibiotics present in the assays could suggest that they affect different toxins. Where kanamycin and cloxacillin are observed to have inhibitory effects in the fibrinogen assay, ampicillin and ampiclox are observed to instead potentiate *C. adamanteus* venom. This can be explained by the antibiotics either affecting different toxins, or affecting the same toxins, but with different effects. In the fibrinogen assay, an inhibiting effect is observed for kanamycin. However, when tested in the fluorescence assay, kanamycin potentiated cleavage. This could theoretically be explained by kanamycin potentiating a protease that has an inhibitory effect on a fibrinogen cleaving protease (e.g., by cleaving this other protease). Another possible explanation for the observed results in the fibrinogen assay is that the antibiotics mainly affect the substrate instead of the snake venom. Possibly, the potentiating effects seen particularly in Figure 1B,D also exhibit a shorter lag phase, which could be explained by a difference in the protease-substrate interaction induced by the antibiotics. This would effectively mean that the increased protease activity is, at least in part, caused by increased substrate availability. Other possible explanations for the inhibitory and potentiating effects observed in this study may serve as the starting point for new hypotheses that in the future can be further explored biochemically or even in the clinical setting.

## 3. Conclusions

In this study, the effects of different commonly used antibiotics on the snake venom protease mediated cleavage of fluorogenic peptide substrate ES011 and fibrinogen to fibrin were observed. Our findings raise an important question regarding the use of antibiotics in the routine management of snakebite envenoming. There is a need to develop efficient assays representative of snakebite envenoming in vitro, and we have developed two assays that could usefully represent an in vivo envenoming scenario. Follow-up studies are needed to better elucidate the utility of these assays and to begin to understand the effect of antibiotics on proteolytic snake venom toxins in humans. Follow-up studies may include in vitro blood coagulation experiments, additional fibrinogen and fluorescence experiments carried out under physiological temperatures, and experiments evaluating whether antibiotics affect the efficacy of antivenom. Thus, this study only fang marks the first bite into a new area of investigation of the interaction between drugs and venom toxins.

## 4. Materials and Methods

Two different assays were employed to assess the effect of selected antibiotics on the enzymatic activities of snake venom proteases. The first assay monitors the cleavage of fibrinogen (F8630-1G, Sigma-Aldrich) to the more turbid fibrin by *C. adamanteus* whole venom (EDB LOT:0617, Kentucky Reptile Zoo). The cleavage was measured as an increase in absorbance at 310 nm by an Epoch spectrophotometer from Biotek (15020518), with associated gen5 software. The antibiotics used in this assay were kanamycin (A1493, 0050, AppliChem), ampicillin (A9518-100G, Sigma-Aldrich), cloxacillin (J64314, Alfa Aesar), and ampiclox (prepared as a 1:1 mixture of ampicillin and cloxacillin). In short, 8 µg/mL of *C. adamanteus* venom was preincubated with varying concentrations of antibiotics for 30 min at room temperature in a 96-well polystyrene microtiter plate (#3370, Costar), before being mixed with 1600 µg/mL fibrinogen for a final volume of 250 µL. Immediately upon addition of fibrinogen, kinetic measurements at 310 nm were initiated and continuously performed each 60th second over the course of 60 min. As negative control, the same procedure was carried out without addition of venom.

The second assay employed was a quenched fluorescent peptide-based substrate assay, which monitors the cleavage of a fluorogenic peptide substrate (Cat#ES011, R&D systems, Minneapolis, MI, USA) that is a known cleavage substrate for different serine proteases involved in the blood coagulation cascade, such as thrombin (Factor II), Factor VII, and a range of kallikreins. Different concentrations of antibiotics were mixed with either 8 µg/mL *C. adamanteus* whole venom, 8 µg/mL *B. asper* whole venom, or with 6 µg/mL of the *C. durissus terrificus* (South American rattlesnake) serine protease, gyroxin [5]. Gyroxin was purified according to Barros et al. [9]. The mixtures were incubated for 30 minutes at room temperature in black 96-well plates (#237105, Nunc). Then, 100 µg/mL of the fluorogenic peptide substrate was added for a final volume of 100 µL, and fluorescence was measured at 85 second intervals over the course of 60 minutes using a SpectraMax iD3 (Excitation 380 nm, Emission 460 nm) from Molecular Devices. Measurements were performed in triplicate, except for two cases, where it was only possible to perform the measurements in duplicate. For the fluorescence assay, pH controls were carried out using 400 µg/mL antibiotics with or without adjustment to pH 7.13 (which was the lowest common denominator for pH), mixed with 8 µg/mL *C. adamanteus* whole venom or phosphate-buffered saline (PBS) with or without adjustment to pH 7.13. For both assays, negative control assays with antibiotics were carried out using the same parameters as in the original assay, but without the addition of venom or gyroxin. 

Venom and gyroxin concentrations were chosen experimentally to ensure a steady signal throughout the experiments. The range of antibiotic concentrations was chosen after studying the literature for snake venom concentrations in blood after a snakebite of either *C. adamanteus* [10] or the similar *C. atrox* (western diamondback rattlesnake) [11]. This was then compared with the antibiotic concentration in blood after a 500 mg oral dose of antibiotics [12,13] to decide what level of antibiotic relative to the venom concentration should be included. One-way analysis of variance (ANOVA) followed by Dunnett’s test was used to determine statistical significance in the different assays. GraphPad Prism software (www.graphpad.com) was used for statistical analysis.

## Supplementary Materials

The following are available online at https://www.mdpi.com/2072-6651/12/4/240/s1. Figure S1: Quenched fluorescent peptide-based substrate assay monitoring cleavage of ES011 by 8 µg/mL *C. adamanteus* whole venom in the presence of 400 µg/mL of either kanamycin (**A**) or ampicillin (**B**) with or without pH adjustment. Figure S2: Quenched fluorescent peptide-based substrate assay monitoring cleavage of ES011 in the presence of three different concentrations of kanamycin (**A**), ampicillin (**B**), cloxacillin (**C**), and ampiclox (**D**). Figure S3: Fibrinogen cleavage assay to test the cleavage of fibrinogen to fibrin in the presence of different concentrations of kanamycin (**A**), ampicillin (**B**), cloxacillin (**C**), and ampiclox (**D**). Figure S4: Quenched fluorescent peptide-based substrate assay monitoring cleavage of ES011 by 8 µg/mL *B. asper* whole venom in the presence of varying concentrations of kanamycin (**A**), ampicillin (**B**).
