# Supplementary material for: Do Antibiotics Potentiate Proteases in Hemotoxic Snake Venoms?
Source: Toxins (Basel). 2020 Apr 9;12(4):240. doi: 10.3390/toxins12040240 (PMC7232225; doi:10.3390/toxins12040240)
Supplement: Supplementary file 1 [file toxins-12-00240-s001.pdf]

Supplementary Materials: Do Antibiotics Potentiate Proteases in Hemotoxic Snake Venoms?

Christoffer V. Sørensen, Cecilie Knudsen, Ulrich auf dem Keller, Konstantinos Kalogeropoulos, Cristina Gutiérrez-Jiménez, Manuela B. Pucca, Eliane C. Arantes, Karla C. F. Bordon and Andreas H. Laustsen

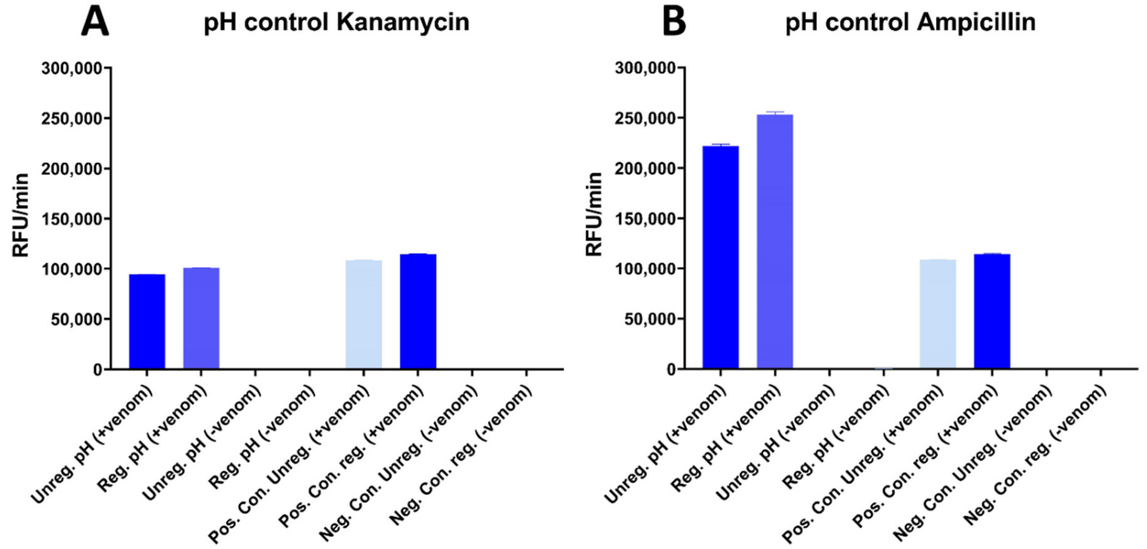

Figure S1. Quenched fluorescent peptide-based substrate assay monitoring cleavage of ES011 by 8 µg/mL *C. adamanteus* whole venom in the presence of 400 µg/mL of either kanamycin (**A**) or ampicillin (**B**) with or without pH adjustment. 'Regulated' indicates that pH was adjusted to 7.13 prior to measurement. Measurements were done in triplicates. RFU: Relative Fluorescence Units. Each column represents the RFU/min calculated using linear regression with error bars showing the standard deviation.

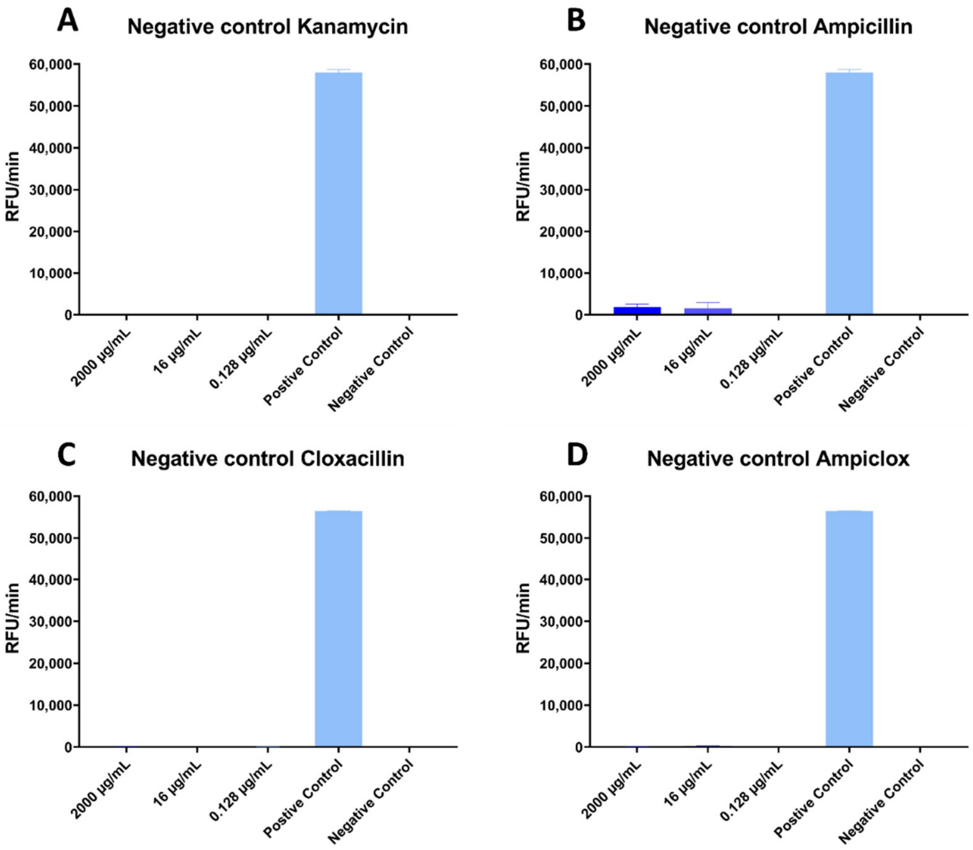

Figure S2. Quenched fluorescent peptide-based substrate assay monitoring cleavage of ES011 in the presence of three different concentrations of kanamycin (**A**), ampicillin (**B**), cloxacillin (**C**), and ampiclox (**D**). 8 µg/mL *C. adamanteus* whole venom was used as positive control and PBS was used as negative control. No antibiotics were added in the controls. Measurements were done in triplicates. Each column represents the RFU/min calculated using linear regression with error bars showing the standard deviation.

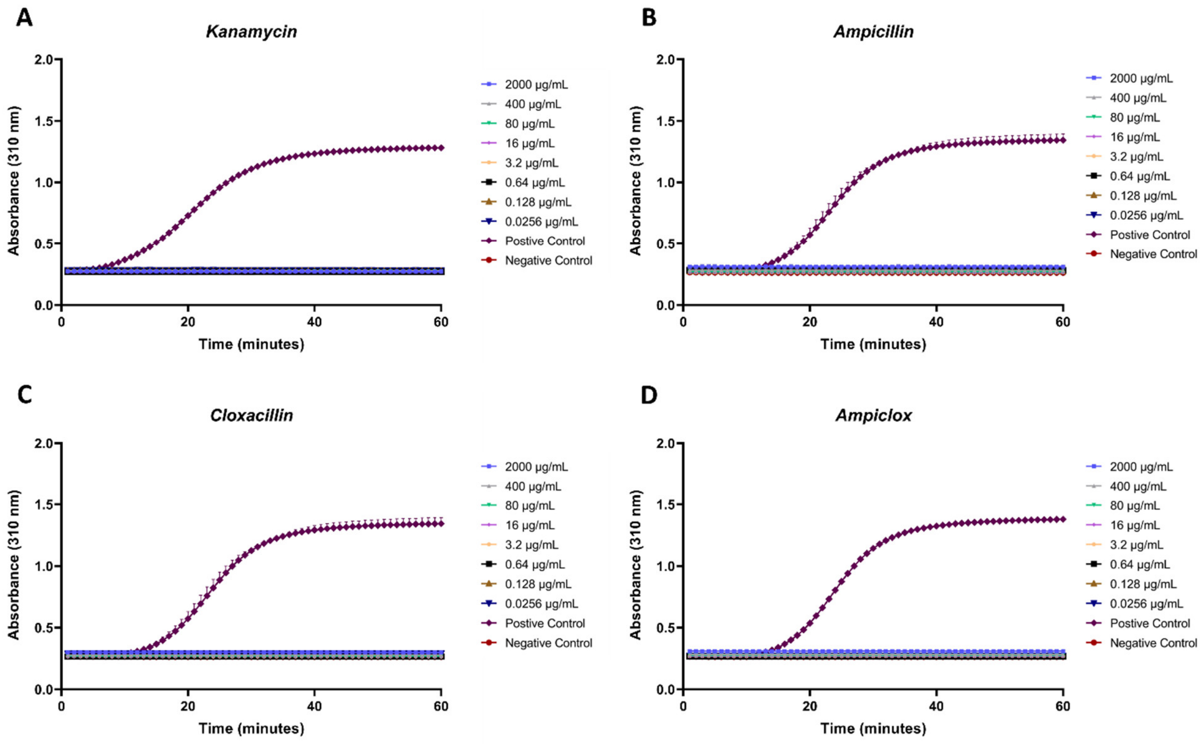

Figure S3. Fibrinogen cleavage assay to test the cleavage of fibrinogen to fibrin in the presence of different concentrations of kanamycin (**A**), ampicillin (**B**), cloxacillin (**C**), and ampiclox (**D**). 8 μg/mL *C. adamanteus* whole venom was used as positive control and PBS was used as negative control. No antibiotics were added in the controls. Measurements were done in triplicates. Each point is the average of triplicates with error bars showing the standard deviation.

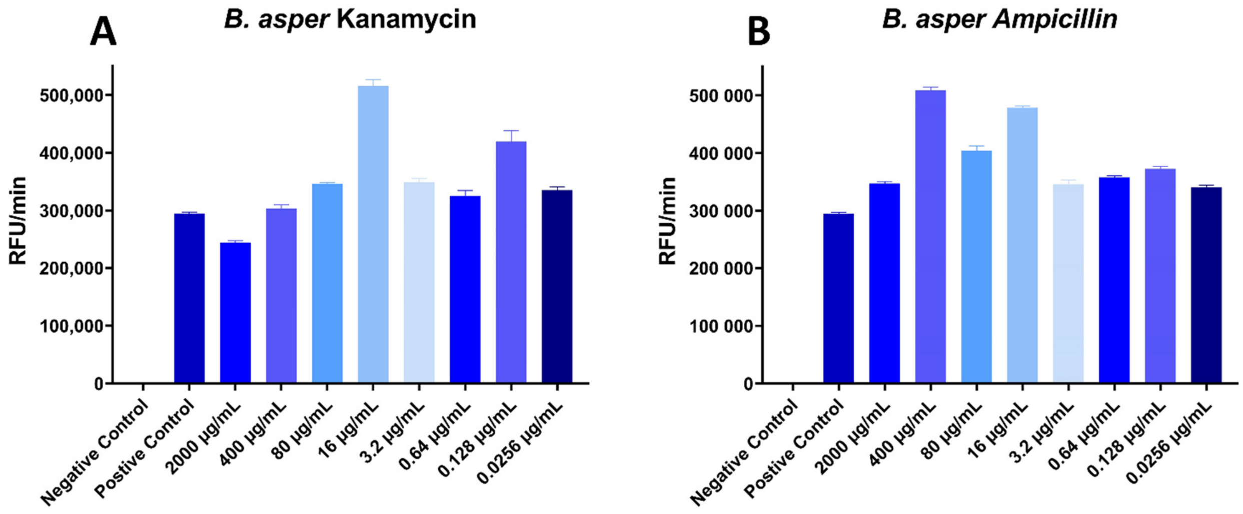

Figure S4. Quenched fluorescent peptide-based substrate assay monitoring cleavage of ES011 by 8 μg/mL *B. asper* whole venom in the presence of varying concentrations of kanamycin (**A**) and ampicillin (**B**). 8 μg/mL *B. asper* whole venom was used as positive control and PBS was used as negative control. No antibiotics were added in the controls. RFU: Relative Fluorescence Units. Measurements were done in triplicates. Each column represents the RFU/min calculated using linear regression with error bars showing the standard deviation.
